# Treatment of Aortic and Iliac Artery Aneurysms with Multilayer Flow Modulator: Single Centre Experiences

**DOI:** 10.1155/2018/7543817

**Published:** 2018-05-31

**Authors:** Cengiz Ovalı, Aykut Şahin, Murat Eroğlu, Sinan Balçın, Sadettin Dernek, Mustafa Behçet Sevin

**Affiliations:** Department of Cardiovascular Surgery, Medical School, Eskisehir Osmangazi University (ESOGU), Eskisehir, Turkey

## Abstract

**Objective:**

Presenting early and midterm results of aortic and iliac artery aneurysms treated with Multilayer Flow Modulators (MFM).

**Methods:**

We retrospectively reviewed the medical records of 23 patients (19 males and 4 females) who are admitted to our clinic between April of 2014 and February of 2016, diagnosed with thoracoabdominal aortic aneurysm and/or iliac aneurysm, and treated using MFM. The patients were followed up for the development of potential clinical presentations for 12 months.

**Results:**

MFM implantation was successfully completed in all the patients. During the process, two patients developed endoleak and so they were treated with postdilatation that was performed through balloon intervention, whereby the patients fully recovered. Although a short-term ischemic cerebrovascular event occurred in one of the patients 36 hours after the MFM, the patient recuperated without any noticeable neurological sequelae. Overall, three patients died after the procedure, one of whom died in hospital three days following the intervention due to acute renal failure, while the second one lost his life at the end of the first month due to the occlusion of superior mesenteric and celiac arteries. The third patient died at the end of the third month due to acute myocardial infarction. The rest of the patients developed no complications or had no mortality at their 12-month follow-ups.

**Conclusion:**

MFM can be preferred as an alternative approach in the treatment of aorta and iliac artery aneurysms including major lateral branches. The present results should be confirmed with additional future studies conducted with larger patient groups for longer periods.

## 1. Introduction

Aneurysms of the aorta and peripheral arteries are uncommon clinical conditions.

Historically, in the literature, the prevalence of thoracoabdominal aortic aneurysm (TAAA) is thought to vary between 1 and 10 per 100,000 people per year [1–4], with less than 7% of all intra-abdominal aneurysms isolated iliac artery aneurysm (IAA). They are found in only 0.03% of the population in autopsy studies [5, 6]. However, up to 40% of iliac arteries aneurysms coexist with Abdominal Aortic Aneurysms (AAA) [5, 6]. If left untreated, they may grow and rupture, ultimately leading to the death of the patient [7, 8].

Aneurysm treatment has been performed with the open surgical approach since the early 1950s [9]. In the last two decades, as a result of the developments of endovascular methods, treatment with percutaneous access has become an alternative to surgery [10, 11]. Endovascular treatment is now the primary option for aneurysm treatment, with regard to its low mortality and risk of morbidity if technically feasible [12–14]. Branches patency must remain a major consideration regarding complex aneurysm treatment. Amongst the well-known adverse consequences of visceral or thoracic aorta branches are bowel infarction, renal failure or necrosis, and paraplegia. Open surgery is thought to cause 13.2% spinal cord ischemia [15–18].

A vast range of endovascular devices have been developed to cope with the presence of major lateral branches within or near the aneurysm sac, the best known of which are the chimney technique, fenestrated stent grafts, and MFM. When the aneurysm is isolated with the chimney technique and fenestrated stent grafts, the flow of the vital lateral branches is maintained mechanically. However, application of these stents is very difficult and the clinical results are unsatisfactory [19]. Notably, the multicentre prospective French study (Windows) on AAA and TAAA treatment with fenestrated and/or branched endovascular repair (f/b-EVAR) [20] reveals up to 16.7% of paraplegia/parapesia for type II and III TAAA, up to 7.1% severe ischemic colitis for suprarenal AAA and type IV TAAA, as well as 2.4% bowel infarction and 23.8% of renal impairment in suprarenal and type II, III, and IV TAAAs. Those complications need to be seriously considered, regarding both the patient's well-being and the medico-economic impact.

MFM is an unconventional endovascular device that has been developed to manage complex aneurysms. MFMs have been shown to be easier to apply and not require a specific preliminary preparation of the prosthesis for each patient in advance, since they are off-the-shelf solution readily available in all size and diameters, with favourable midterm clinical outcome [21–26].

MFMs are uncoated, self-expanding, 3D braided multilayer devices, with high radial force and made of a memory-shape cobalt/chrome alloy (Phynox™). The device is available in a wide range of lengths and diameters, making it possible to treat all locations, shape, and size of aneurysm. It is currently CE marked for peripheral and aortic aneurysms and is under investigation for the treatment of chronic type B aortic dissection. Being porous stents, these multilayered devices modulate the flow dynamics along the vessel wall into the aneurysm, converting recirculating flow into the aneurysm into a laminar flow and at the same time acting as a passive barrier by reducing the flow velocity (>90%); the reduction of flow velocity into the aneurysm, as well as lamination, induces the formation of gradual thrombus in the form of Zahn lines within the aneurysm sac.

On the other hand, since it does not cause any discontinuation of the flow on the side, the MFM redirects the blood flow to the lateral branches due to the Venturi effect [23, 24]. Branches are kept patent when the device is landed before the ostium of emerging arteries. A Systematic Review and Patient-Level Meta-Analysis of the Streamliner Multilayer Flow Modulator in the Management of Complex Thoracoabdominal Aortic Pathology reports a rate of 97.8% of branches patency at the latest follow-up documented in 13 reviewed publications. The French STRATO trial also reports the same range of patency, with 100.0% and 97.0% of branches patency at, respectively, 24 and 36 months [25, 26]. In our study, we provided early and midterm results of patients enrolled and treated with MFMs.

## 2. Methods

After obtaining the approval of Intuitional Ethics Committee, the medical records and computerized data of 78 patients admitted to our clinic between April of 2014 and February of 2016 and received stent graft implantation through endovascular approach owing to aortic aneurysm and iliac artery aneurysm were retrospectively reviewed. Of the 78 patients, 23 patients (19 males and 4 females) who were treated with MFM stents were included in the present study. The common symptom seen in half of the patients was abdominal and back pains. One of the patients showed claudication which became apparent during walking. The rest of the patients were asymptomatic.

Of 23 patients operated, 17 had TAAA while five of them had TAAA associated with bilateral iliac artery aneurysms. Another patient was detected with giant pseudoaneurysm of right iliac artery developed iatrogenically during the surgery for lumbar disc hernia. All the patients had both major lateral branches emerging from the aneurysm sac and showed involvement of the major lateral branches. All the aneurisms were fusiform in shape, apart from the saccular iliac aneurisms. When the patients were evaluated according to aetiology, there was at least one more disease accompanied with nonspecific degenerative aneurysms. Furthermore, 13 patients were inflicted with hypertension. Eleven of them had dyslipidemia. Eight patients were identified to have diabetes mellitus while seven patients had coronary artery disease. Seven patients suffered from chronic obstructive pulmonary disease while four patients had congestive heart failure and peripheral atherosclerotic disease. Three patients were inflicted with renal insufficiency. The maximal diameter of aneurisms in TAAA was between 55 and 76 mm and it was between 34 and 48 mm in IAA patients. The main characteristics of patients have been presented in Table 1.

All the patients who were to receive endovascular intervention due to TAAA and IAA were designated to get contrast-enhanced computed tomography (CT) angiography with 1.5 cm CT section thickness prior to the procedure in our clinic. Structure of the aneurism and the critical branches exiting from it were carefully assessed in detail in CT angiography. Aortic diameter, calcification in the wall of the aorta, presence of thrombus in the vascular lumen, and the length and angulation of the aneurism in which the stent was to be placed were evaluated and measured as well. Moreover, the type and dimensions of the grafts that were to be used were also evaluated with regard to their compliance with the size of the iliac and femoral arteries. Diameter of the graft was determined using 20% larger of the diameter that was measured at the proximal and distal ends of the graft that were to be placed to healthy part of the vessel.

Endovascular attempt was preferred since planned surgical interventions had high risk of mortality and morbidities. Surgical interventions were performed in the angiography unit after obtaining written consent of the patients and implementing required sterilization of the operation room. The surgical interventions were completed under local anaesthesia accompanied with sedation in 21 patients and under general anaesthesia accomplished through femoral artery in 2 patients. Cardiatis MFM stents were used throughout implantation procedures in 23 patients.

Effective implantation of MFM within the aneurysmal area without complications was defined as a successful intervention. Inclusion criteria were as follows:

- Patient suitable for endovascular repair (EVAR) or thoracic endovascular repair (TEVAR) for thoracoabdominal aneurysms and not suitable for graft stent regarding the presence of iliac artery aneurysms.
- 55 mm or more for thoracoabdominal aneurysms diameter and presence of giant aneurysm (>35 mm) affecting internal iliac artery for iliac artery aneurysms.
- Presence of major lateral branches within or near the aneurysm incision
- Patients technically suitable for MFM implantation (<60 degrees proximal angulations, aneurysm diameter < 90 mm, distal and proximal landing zone > 20 mm, and iliac artery diameter > 6.5 mm)

The patients were monitored for 48 hours, being released with the daily treatment of 100 mg aspirin and 75 mg clopidogrel. The patients were checked at postoperative 1, 3, 6, and 12 months to assess conditions of the endovascular stents. In addition, all the patients received a CT angiography at postoperative 3rd month to further evaluate condition of the endovascular stents.

## 3. Results

**Figure 1:**
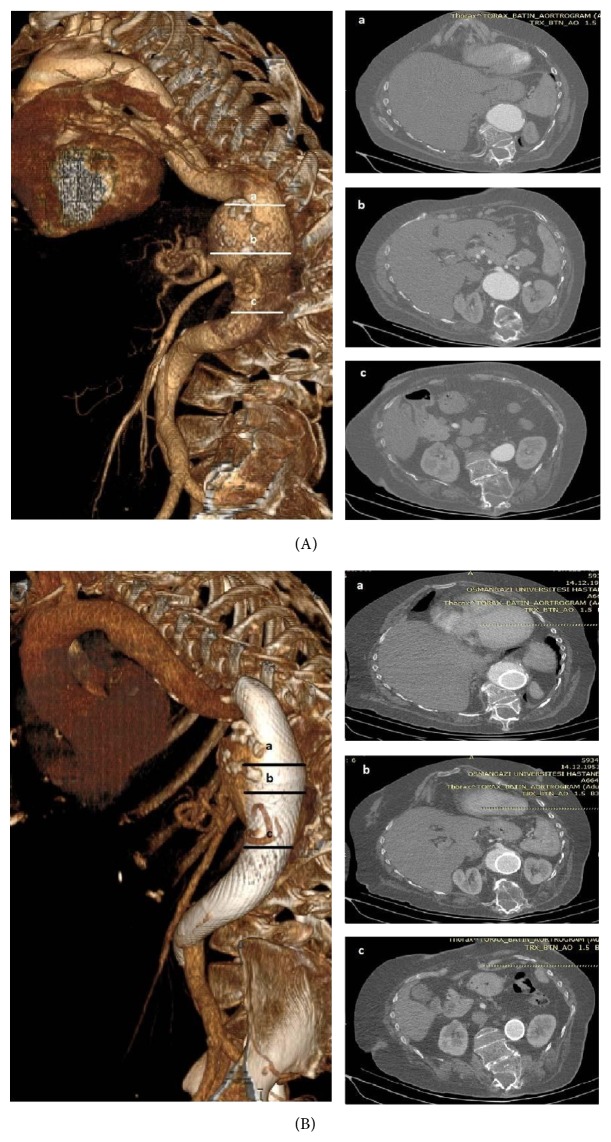
(A) Images of the patient with thoracoabdominal aortic aneurysm. (B) Images of the patient with thoracoabdominal aortic aneurysm after the multilayer flow modulator device application.

**Figure 2:**
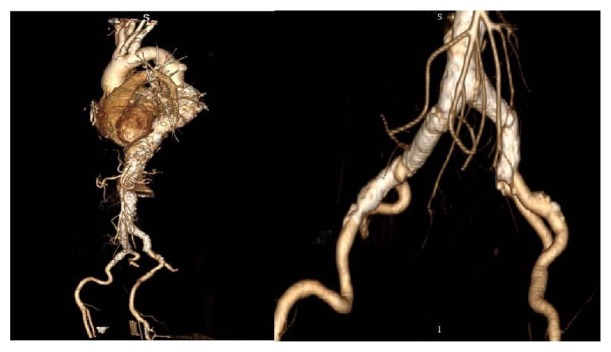
Female patient with advanced calcifications (porcelain aorta).

The procedure was successfully completed in 23 patients (Figures 1(A) and 1(B)) but two patients were excluded from the current study since the delivery system failed due to extensive calcifications and/or access artery tortuosity. In two patients (one male and one female), the reason for failure was that the delivery system could not be advanced to the target area. In the female patient, advanced calcifications (porcelain aorta) are the reason for the inability to pass through the introducer femoral artery (Figure 2). The left subclavian artery was the surgically cut down to provide new access, but the introducer could not be advanced there. Similarly, in the other patient, the sheath could not be advanced due to excessive calcification and tortuosity in iliac arteries. It is to note that inadequate arterial access (due to tortuosity or calcifications) is presently contraindications for MFM implantation.

All the operations were planned to be performed under local anaesthesia and deep sedation. However, since one patient refused to be treated under local anaesthesia and one other presented with excessive anxiety, both were treated under general anaesthesia. The remaining 21 patients were treated with local anaesthesia. The procedure was successfully implemented in all the patients. The device was introduced through a surgical cutdown of the common femoral artery in 22 patients, and one patient (with iliac aneurysms) underwent a percutaneous approach. Only two patients developed type 1 endoleak as procedural complication. Following control angiography, the proximal part of the MFM was dilated with an extension balloon of 46 mm, thus eliminating the endoleak. No further complications, such as MFM migration and no aneurysm rupture, were noted. A total of 45 MFM were used to treat a total of 23 patients, with a mean number of 2 MFM per patient. In three patients, postdilation procedure was performed because the MFM had not achieved a desirable opening. The average duration of the intervention was 65 minutes. Intervention characteristics have been summarized in Table 2.

**Figure 3:**
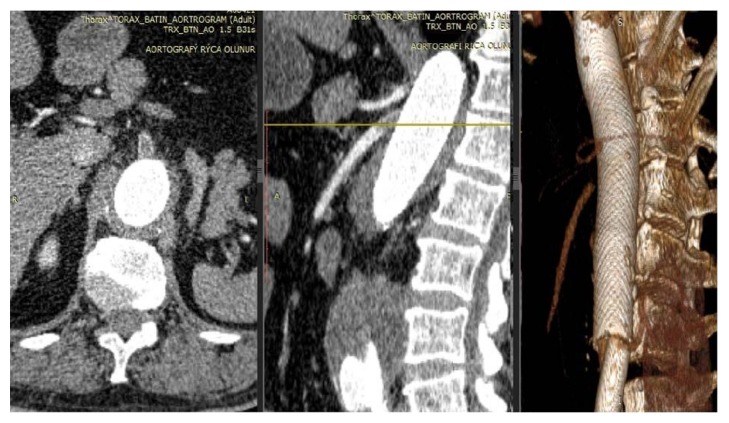
Images of CT angiography which showed the occlusion of SMA and the Coeliac due to thrombus.

Patients were followed for a total of 12 months in terms of clinical event development. One patient (70 years old male) died in hospital on the third day due to acute renal failure (ARF), another one dying (72 years old male) at the end of the first month due to Superior Mesenteric Artery (SMA) and Celiac Artery (CA) occlusion. Still another (78 years old male) died at the end of the third month due to acute anterior lobe myocardial infarction (MI). It was assumed that secondary nephropathy, induced by the contrast agent in the patient who died in the hospital and hypotension, developed during the procedure. Doppler ultrasonography revealed that both renal arteries were patent in this patient. This patient was hemodialyzed twice and died on the 5th day of his follow-up. The patient who died at the end of the first month presented with a type II TAAA and implanted with 2 MFM. This patient was advised to take clopidogrel 75 mg/day and ASA 100 mg/day for 6 months. It has been learned, however, that the patient had not taken his dual antiplatelet therapy the week before. CT angiography showed that the SMA and the Coeliac Trunk were occluded by intense thrombus load (Figure 3). The patient was urgently operated upon but died two days after the reintervention. The third death was caused by an acute anterior MI recurrence at the end of the third month. The patient subsequently died from acute cardiogenic shock.

Within the hospitalization period, a short ischemic cerebrovascular event occurred 36 hours after implantation of two MFM into a type II TAAA presented by a 63-year-old patient. There were no neurological sequelae and this patient continued the dual antiplatelet therapy for 12 months. A 78-year-old patient with an implanted MFM due to type III TAAA developed contrast-induced nephropathy. This patient did not require haemodialysis and his renal function returned to normal by the end of the 7th day. In-hospital and 12-month unwanted clinical events are summarized in Table 3.

## 4. Discussion

In the current study, we present the early and midterm results associated with the usage of MFM, which is used in treating the patients diagnosed with TAAA and IAA. While the procedure was successfully in 23 patients, it was unsuccessful in two patients due to calcification and tortuosity in the iliofemoral artery, which hindered advancement of the delivery system into the aorta, so these two patients were excluded from the study.

Both aorta and peripheral arteries aneurysms can grow with time. With the increase in aneurysm diameter, the radial stress upon the aneurysm wall increases as well. At the same time, recirculating flow into the aneurysm sac is amongst the most important factors that lead to further expansion of aneurysm and its rupture [27, 28], inducing a profound shift of the artery wall towards elastolysis, inflammation, and oxidative stress. Of all the suggested methods for treating aneurysms, it is endovascular methods that are regarded as the golden treatment approach. The purpose of all endovascular treatments is to prevent aneurysm rupture, almost lethal in every case [29, 30]. The choice of EVAR technique must be customized depending on the patient and aneurysm presentation. Stent graft is currently the most common choice for endovascular treatment of aneurysms with no major branches involved. However, there is still no definite and completely off-the-shelf treatment for aortic aneurysms involving major branches.

All the patients included in this study had a significant number of major branches involved (total of 60 branches). While custom-made fenestrated stent grafts were used in some studies of TAAA with branches, the multilayer flow modulator has also been used in such pathologies [21–26].

MFMs are relatively newly developed devices. They have been CE marked for the treatment of peripheral aneurysms since 2009 and since 2011 for aortic aneurysms. In our single centre study, the MFM was used to treat all our patients. Although preliminary results are satisfactory, there is a need for long-term follow-up and a stronger clinical experience.

The MFM may provide valuable solution in the treatment of aneurysms when patient selection is performed accurately. In a meta-analysis evaluation performed on seven studies with a total of 155 patients treated with custom-made branched/fenestrated stent grafts, mortality was 7.1% at 30 days and 16.1% through a mean follow-up of 11.8 months [31]. In addition, 30-day mortality was 0.0% in the STRATO study, which was initiated in 2010 and was performed on 23 patients. But one patient died during the first year of follow-up [25]. The all-cause mortality rate at the end of the first year of follow-up was found to be 5.5% with one perioperative death, all deaths being not aneurysm-related in another multicentre registry study involving 54 patients whose peripheral and visceral arteries were treated with the MFM [32]. The aneurysm rupture rate, as documented in a meta-analysis performed on 15 MFM-specific publications, was reported as low as 1.2%. Importantly, all ruptures occurred in cases probably out of Instruction For Use, as quoted by the authors [26].

MFM devices trigger the formation—in a controlled way—of an organized thrombus within the aneurysm without leading to any degradation in flow patency in collaterals. Furthermore, the aneurysm gets stabilized with time. The porosity of the device, inducing a drop of pressure and an increase in blood flow velocity as it gets through the mesh, ensures that the branches remain patent [24, 32]. Our results are consistent with those presented in the MFM-specific literature. When the clinical results of the studies regarding this topic are taken into consideration, in the event where significant subbranches emerge out of the aneurysms or are located very nearby and in the device landing zone, considering MFM first may be reasonable.

In our study, three patients passed away by the end of month 12. The first patient who passed away in our study was lost on acute renal failure (ARF) 5 days after MFM implantation. A total volume of 230 ml of contrast agent was used on this patient during the operation. The duration of the operation was 110 minutes. Before operation GFR was 52 ml/min. Both renal arteries were documented patent before and after the operation. We think that ARF developed in this patient due to a contrast nephropathy. ARF related to contrast nephropathy is a complication that could potentially be observed in such group of high risk, polymorbid patients. The most important causes of contrast nephropathy are the amount of the contrast substances used, basal renal functions, coronary failure, and diabetes mellitus [33].

Most of the patients had moderate renal failure and type 2 diabetes mellitus and were at high risk of contrast-induced nephropathy: the amount of contrast agent must remain minimal. Also, intravenous serum physiological liquid at a speed of 1 ml/kg/hour including the 12 hours before the operation and another 12 hours afterwards must be given. During the operation, particular care should be observed in order not to leave the patient hypotensive. All those precautions were taken in this series of patients; however, endovascular intervention by its nature may be harder and more complex than planned. Similarly, in the patient passing away due to possible contrast-induced nephropathy, the duration of operation and the amount of contrast were higher than expected. We think that hypotensive fluctuations happening a few times during the operation caused hypoperfusion and contributed to the severity of the contrast-induced nephropathy.

The second patient who passed away in our study suffered from SMA and CA thrombosis by the end of the 30th day of follow-up. In the medical record obtained from this patient, it was found out that he had not been taking the recommended dual antiplatelet treatment for the last three weeks of the follow-up. In the CT angiography examination of the patient, SMA and CA were occluded due to thrombosis (Figure 3). Surgical therapy was performed. On the second day following the reintervention, his general situation deteriorated and he passed away.

Aortography records related to MFM implantation in this patient were examined again. In the presentations before and after the operation, it was observed that subbranch ostia were open. We think that the subacute thrombosis condition that developed in this patient is indeed due to lack of dual antiplatelet treatment. As is the case in our patient, in patients whose significant subbranches are covered by the device, we think it is necessary to get the dual antiplatelet treatment in long term. Unfortunately, there is not a consensus regarding the intensity and duration of the antiplatelet treatment that is to be performed in these patients [25, 34, 35]. In a study carried out by Pane et al. dual antiplatelet treatment following MFM was used for 12 months for TAA aneurysm [19]. However, one should remain careful when using dual antiplatelet since the stabilization of the aneurysm process induced by the MFM relies on the formation of an organized thrombus [24].

The other highly risky group of patients in terms of thrombosis development is the group presenting with atheromatous plaque causing a >50–70% stenosis. If this is the case, the decision to get treatment must be taken more carefully, and it must be considered to perform percutaneous treatment of the stenosed branches before endovascular treatment of aneurysm in required situations [31]. In our centre, we advise our patients to get dual antiplatelet treatment (clopidogrel 75 mg/day and ASA 100 mg/day) for minimum 12 months and ASA 100 mg/day and single antiplatelet treatment indefinitely for afterwards.

The third patient that passed away in our study suffered from an acute anterior MI (MI). In this group of patients, the frequency of coronary artery disease is quite high. 33% of the patients in our study population presented with coronary artery disease. Therefore, this group of patients is at high risk for developing MI. While planning the treatment of these patients, it must also be ensured that they get an optimal treatment for coronary artery disease [35, 36]. In conclusion, none of the three deaths was MFM related and only one death was procedure related (the patient who suffered from kidney insult following the use of contrast agent). The MFM can be regarded safe and performant for the treatment of aneurysms involving significant subbranches.

Moreover, since we failed to get an iliofemoral access, endovascular intervention was not performed in two patients who were excluded from the study. The most important reason of the impossibility of the MFM delivery system to be advanced through the access is that the size of artery is not sufficient or the access artery presents calcification and tortuosity [37]. The fact that there is an excessive amount of tortuosity in the vein is another problem that makes it difficult to intervene percutaneously. When there is tortuosity, the vein may be flattened by using hard wires with assistance force. This assistance force might be increased with two or three wires when necessary. Importantly, patients presenting with such tortuous or calcified access arteries are contra-indicated per the current MFM Instruction for Use.

## 5. Limitations

We are aware of the fact that the present study has some limitations. The current study was a retrospective one and provided results of short follow-up regarding the competence of the MFM in the treatment of aorta and iliac artery aneurysms with major branches involved.

## 6. Conclusion

The present results suggest that MFM is safe and reliable and can be preferred as an alternative approach in the treatment of aorta and iliac artery aneurysms with major branches involved. Future studies with not only larger patient population but also longer period of follow-up are needed to further confirm and strengthen the current results.

## Figures and Tables

**Table 1:** Demographic characteristics of the patients and lesion features.

	*n*	%
Age (year) (min–max)	72,5 (55–81)	
Gender (male/female)	19/4	
HT	13	56
Dyslipidemia	11	47
Renal insufficiency	3	13
Smoking	14	60
DM	8	35
COPD	7	30
CHF	4	17
CAD	7	30
Peripheral atherosclerosis	4	17
Max diameter of aneurysm (mm)	*Mean*	*(Min*–*max)*
(i) Aorta	58.6	(55–76)
(ii) Iliac	36.5	(34–48)
Aneurysm length (mm)		
(i) Aorta	132	
(ii) Iliac	36	
Thoracoabdominal aneurysm type [22]	*n*	%
(i) Crawford type 1	5	23
(ii) Crawford type 2	4	18
(iii) Crawford type 3	7	32
(iv) Crawford type 4	6	27
Lateral branches asset	23	100

CAD: coronary artery disease, CHF: chronic heart failure, COPD: chronic obstructive pulmonary disease, DM: diabetes mellitus, and HT: hypertension.

**Table 2:** Intervention-related results.

	*n*	%
Type of anaesthesia		
(i) Local	21	19
(ii) General	2	9
Number of MFM		
(i) 1	6	26
(ii) 2	12	52
(iii) 3	5	22
Postintervention-related complications		
(i) Endoleak	2	9
(ii) Femoral hematoma	-	
(iii) Device migration	-	
(iv) Aneurysm rupture	-	
Postdilatation	3	13
Intervention success	23	100
Intervention time (minutes)	65 ± 23 (43–115)	
Hospitalization duration (days)	5.6 ± 2.3 (3–10)	

MFM: multilayer flow modulator.

**Table 3:** Undesired clinical events during in-hospital treatment and 12-month follow-up period.

	In-hospital period	12 month
MI (*n*)	-	1
CVE (*n*)	1	-
Acute renal failure (*n*)	2	-
Occlusion of lateral branch (*n*)	-	1
Aneurysm rupture (*n*)	-	-
Deaths (*n*)	1	2

CVE: cerebrovascular event, MI: myocardial infarction.
